# A case of severe endogenous endophthalmitis with orbital cellulitis post COVID-19

**DOI:** 10.4102/aveh.v81i1.748

**Published:** 2022-09-30

**Authors:** Herold L. Letsoalo, Solani D. Mathebula

**Affiliations:** 1 Department of Ophthalmology, Botshilu Private Hospital, Ga-Rankuwa, South Africa; 2 Department of Optometry, Faculty of Health Sciences, University of Limpopo, Sovenga, South Africa

**Keywords:** 2019 coronavirus, corticosteroids, COVID-19, endogenous endophthalmitis, orbital cellulitis

## Abstract

Coronavirus disease 2019 (COVID-19) is a novel pandemic viral illness which may lead to severe respiratory diseases. However, its ophthalmic manifestations are still not well understood. There could be serious complications involving various organs in patients with the disease and in those who are recovering, or have recovered, from COVID-19. Endogenous endophthalmitis, was a rare occurrence in the past but has increased in the post-COVID-19 pandemic. Endogenous endophthalmitis results from hematogenous spread from a remote primary source. A 55-year-old man with confirmed COVID-19 presented to a local hospital with unilateral gradual loss of vision and swelling of the left eye that occurred 10 days after discharge from the hospital. Based on detailed ophthalmic examination, this patient with a co-morbidity, namely diabetes mellitus was placed in the intensive care unit where he was given corticosteroids and ventilation for two weeks. Most patients requiring mechanical ventilation may experience disorders of the eye, and it may be difficult to treat these occurrences while the patient remains in ICU. COVID-19 can lead to ocular complications especially in the late stages of infection. Follow-up of post COVID-19 patients is crucial to monitor possible ocular complaints including endophthalmitis as early diagnosis and treatment is essential to salvage vision.

**Contribution:**

There is a possible link between COVID-19 and endophthalmitis, which is a rare but devastating eye infection. The medical community should consider the eye when evaluating the role of anti-infectious treatment and immunomodulation.

## Introduction

Since the outbreak or the emergence of the novel coronavirus diseases (COVID-19) in December 2019, there have been unparalleled global efforts to characterise the virus.^1,2,3^ The infectious agent of COVID-19, which causes severe acute respiratory syndrome, is the severe acute respiratory syndrome coronavirus 2 (SARS-CoV-2), an enveloped *beta coronavirus* with a genetic sequence very similar to SARS-CoV-1.^1,2,3^ COVID-19 is a highly contagious diseases^1^ that follows a biphasic pattern of illness resulting from the combination of an early viral response phase and an inflammatory second phase.^2^ COVID-19 is mainly transmitted between people through direct contact or respiratory droplets from an infected person when coughing or sneezing.^2^ Prior to SARS-CoV-2, two other human coronaviruses emerged, which caused respiratory failure or influenza-like illness.^1,4^ SARS-CoV-2 affected many previously healthy individuals who developed severe pneumonia with rapid oxygen desaturation requiring urgent intensive care unit (ICU) hospitalisation for respiratory support, intravenous drugs, corticosteroids, and fluids.^5^ All of these could predispose healthy individuals to secondary infections.

COVID-19 had been previously reported to be associated with mild conjunctivitis and increased tearing which are indistinguishable from other viral conjunctivitis in humans.^6,7,8,9,10,11^ However, ocular complications have not been widely reported and the viral loads in ocular tissue are yet to be investigated. The purpose of this article is to document the presentation of unilateral endogenous endophthalmitis combined with orbital cellulitis in a patient that occurred post COVID-19. Endogenous endophthalmitis with or without orbital cellulitis is a rare entity but such cases may be on the rise post-COVID-19 infection. The findings of this case study may facilitate the understanding of ophthalmic complications in patients with COVID-19.

## Case report

A 55-year-old man presented to Botshilu Private Hospital with a 4-day history of painful eye associated with a gradual loss of vision in the left eye. There was also redness and swelling of the eye. The patient also had diabetes mellitus type 2 and was using metformin 850 mg twice orally and diamicron 80 mg twice orally. The patient was previously admitted for severe pneumonia from SARS-CoV-2 and was in an ICU, where he was given high-dose steroids and ventilation for two weeks. After discharge, 10 days later, the patient presented to the hospital emergency department. There was no history of ocular injury or surgery, and his medical history was unremarkable prior to COVID-19, except for diabetes. The patient was clinically stable, no longer having pneumonia.

**FIGURE 1:**
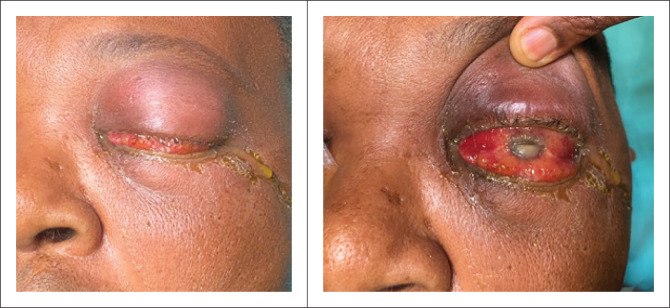
Clinical picture of the left eye of the patient with endogenous endophthalmitis and orbital cellulitis. Moderate-to-severe swelling of the upper and lower eyelids can be noted.

Medical examination revealed a temperature of 38.2 °C with a pulse rate of 110 beats/min. On ocular examination, the visual acuity in the right eye was 6/6 and in the left eye no light perception. The left eye was immobile with moderate proptosis and swollen periorbital area on the left side. There was severe conjunctival hyperaemia with corneal opacity involving the left eye (see Figure 1). There was also a 40% left hypopyon with severe flare and grade 4+ cells in the left anterior chamber according to the Standardisation of Uveitis Nomenclature grading.^12^ Further details could not be identified. The right eye and right side were normal with no uveitis or retinopathy. An ultrasound B scan was done on the left side and demonstrated vitreous opacity. A diagnosis of left eye endogenous endophthalmitis was made, and samples of blood were sent for microbiological examination.

The patient was admitted immediately, and computed tomography scan of the brain and orbit was performed, but no orbital or brain abscess was identified. Blood cultures were negative after five days. Blood tests confirmed SARS-CoV-2 being positive after 22 days, and serum C-reactive protein was 102 mg/L, which was very high. The urea and electrolytes were normal. The estimated glomerular filtration rate was also normal.

The physician who previously treated the patient in the ICU was consulted to help with the management of other comorbidities. The patient was started on intravenous antibiotics and some anti-inflammatories; also, the patient was initially counselled on removal of the left eye, and an evisceration was proposed as the definitive procedure. After 48 h of treatment with no improvement, the patient was finally prepared for surgery and an evisceration was done in the theatre. A lot of pus was drained.

A specimen was sent for microscopy, culture and sensitivity. A silicon ball orbital implant was inserted after an intense washout with betadine and copious amount of water. On day 5 after surgery, the patient and orbital cellulitis had improved significantly, and the patient was discharged on day 6 with oral antibiotics and anti-inflammatories. Ciprobay and xefo were given as oral treatments to take home for another 10 days. Ciprobay as an antibiotic of choice was because of culture and sensitivity results, as the patient had *Pseudomonas aeruginosa*. After six weeks, the patient came back and the socket was aseptic, the swelling of the eyelids had subsided and thus he was referred to an ocularist for an eye prosthesis. One month later, the patient came to thank the staff and was well-adjusted and satisfied with the eye prosthesis.

## Discussion

Although COVID-19 has many ocular manifestations, endogenous endophthalmitis is a rare or less reported complication. The most common risks of COVID-19 infection include diabetes mellitus, malignancy, immunosuppression and prolonged use of corticosteroids.^13,14,15^ The exact pathogenic mechanisms of the ocular infections because of COVID-19 are still relatively unknown. COVID-19 infection requires treatment with high doses of systemic steroids and immunomodulatory drugs, along with systemic comorbidities, such as diabetes mellitus, which could make such patients immunocompromised, thereby reactivating many latent infections in the body and possibly causing endogenous endophthalmitis.

The patient was a known diabetic and presented with complaints of diminution of vision and pain in the left eye after 10 days post-COVID-19 infection. As the patient had vitreous exudates, no history of uveitis, no intraocular trauma or surgery and vitreous sample culture, a diagnosis of endogenous endophthalmitis was made based on these clinical entities. Endogenous endophthalmitis is a very severe sight-threatening ocular infection presenting as a potential ocular emergency.^13,14,15^ The diagnosis is based on clinical signs, such as the presence of both anterior and posterior segment inflammation, vitreous exudates and absence of any potential exogenous causes, such as ocular trauma or surgery. As far as we know, this is the first case of endogenous endophthalmitis reported in the literature with *P. aeruginosa*.

Patients undergoing treatment for COVID-19 infection with a history of hospitalisation, ICU stay and prolonged systemic corticosteroids with c-morbidities like diabetes mellitus may be prone to endogenous endophthalmitis, both bacterial and fungal.^16,17^ However, the ultimate outcome depends on the virulence and load of the microbial organism causing the endogenous endophthalmitis.

Acute respiratory distress syndrome is a life-threatening condition which requires respiratory support in the ICU.^16,17,18^ Patients admitted to the ICU for COVID-19 because of their comorbidities for respiratory support are also at high risk of suffering from ocular hypoperfusion and may develop ocular complications, such as ocular surface disorders, intraocular pressure elevation, cataract, and other anterior and posterior segment disorders.^17^ The incidence of ocular complications in patients in the ICU varies from 3% to 60%, although sight-threatening complications are rare. Intensive care unit staff, however, must be aware of such ocular complications, and visual or ocular complaints in COVID-19 inpatients should be assessed very promptly.

The most common ocular manifestation of COVID-19 infection is conjunctivitis.^6,7,8,9,10,11^ The most reported symptoms include eye redness, itching, foreign body sensation, photophobia and blurry vision. However, the exact incidence of ocular involvement in patients with COVID-19 is still unclear, ranging between 0.0% and 31.6%.^8^ In patients with conjunctivitis, the virus may be present in the tear layer and could be detected in conjunctival swab samples using reverse transcription polymerase chain reaction (RT-PCR).^18,19^ In the patient reported in this study, the PCR was not done as the patient had already started systemic corticosteroid therapy. Bertoli et al.^19^ reported that there is usually a low percentage of positive results when PCR is performed after the initiation of steroids. It is possible that in negative cases the viral load is below the threshold for positive test detection.

Generally, the combination of endogenous endophthalmitis and orbital cellulitis is very rare. However, in this patient this combination was the first presenting sign. The origin is unknown but could result from metastatic spread of the organism from a primary site of infection in the setting of bacteria or fungemia.^20^ Endogenous endophthalmitis is often related to underlying systemic risk factors, including recent hospitalisation, diabetes mellitus and immunosuppression. In such instances, there may be generalised lowering of immunity causing endophthalmitis and orbital cellulitis as in this patient.

## Conclusion

This case report highlights the need to further understand the full spectrum of ocular complications associated with COVID-19 infection. All medical personnel should have a high index of suspicion of endogenous endophthalmitis and orbital cellulitis in post-COVID-19 patients complaining of blurred vision, pain, swelling and redness around and/or within the eye. Early detection and prompt treatment is the key to controlling the infection and preserving vision. Patients receiving high-dose corticosteroids in the ICU for COVID-19 infection should have comprehensive and regular ocular examinations. While steroids are life-saving medications in patients with severe COVID-19, they can predispose patients to secondary infection with organisms, such as *P. aeruginosa*.
